# Three-Dimensional Bone-Image Synthesis with Generative Adversarial Networks

**DOI:** 10.3390/jimaging10120318

**Published:** 2024-12-11

**Authors:** Christoph Angermann, Johannes Bereiter-Payr, Kerstin Stock, Gerald Degenhart, Markus Haltmeier

**Affiliations:** 1 VASCage—Centre on Clinical Stroke Research, Adamgasse 23, A-6020 Innsbruck, Austria; 2 Core Facility Micro-CT, University Clinic for Radiology, Anichstraße 35, A-6020 Innsbruck, Austria; 3 Department of Orthopedics and Traumatology, Anichstraße 35, A-6020 Innsbruck, Austria; 4 Department of Mathematics, Universität Innsbruck, Technikerstraße 13, A-6020 Innsbruck, Austria

**Keywords:** bone micro-architecture, medical image synthesis, generative adversarial network, StyleGAN, GAN inversion

## Abstract

Medical image processing has been highlighted as an area where deep-learning-based models have the greatest potential. However, in the medical field, in particular, problems of data availability and privacy are hampering research progress and, thus, rapid implementation in clinical routine. The generation of synthetic data not only ensures privacy but also allows the drawing of new patients with specific characteristics, enabling the development of data-driven models on a much larger scale. This work demonstrates that three-dimensional generative adversarial networks (GANs) can be efficiently trained to generate high-resolution medical volumes with finely detailed voxel-based architectures. In addition, GAN inversion is successfully implemented for the three-dimensional setting and used for extensive research on model interpretability and applications such as image morphing, attribute editing, and style mixing. The results are comprehensively validated on a database of three-dimensional HR-pQCT instances representing the bone micro-architecture of the distal radius.

## 1. Introduction

The adoption of deep learning (DL) into the broad field of medical imaging is an ongoing and remarkable success story. From decision support systems in radiology [1] and over-segmentation algorithms for complex organ and tumor regions [2,3] to applications for image enhancement and super-resolution [4], the use of learning-based techniques has led to many advances with great potential for future applications. Such applications require the availability of large amounts of training data to ensure a sufficient range of population variability and thus to increase the reliability of the developed models [5]. When it comes to the development of medical applications, the availability of sufficient data in the relevant modalities is often limited. In addition, sharing medical data with other institutions or even between different hospitals is a major challenge for legal and privacy reasons [6]. These limitations make it challenging to integrate existing modern methods into routine clinical practice.

### 1.1. Generative Modeling

A promising approach to overcome the above-mentioned challenges is the synthetic generation of realistic targeted data samples. This not only ensures patient privacy but also allows new types of images with specific characteristics to be synthesized on demand, enabling medical research on a much larger scale. Within the field of generative modeling, the advent of generative adversarial networks (GANs) in 2014 can be seen as a major catalyst [7,8]. GANs have significantly advanced a wide range of life-science applications [5,9] as well as other areas within medical imaging, including modality transfer [10,11] and image segmentation [12]. Generative models approximate the probability density function underlying the available data and can thus produce realistic representations of examples that differ from those in the training data [13]. GANs have achieved remarkable improvements in the quality of natural images [14,15], and also allow for good control of output diversity and resolution. In addition, the introduction of GAN inversion techniques has allowed a variety of new possibilities beyond synthesis, such as attribute manipulation, image transitions, and style mixing, to name a few [16].

A major challenge in using generative models for medical applications is the dimensionality of the data. Existing GANs are mainly built and tested on large datasets of two-dimensional images, such as the CelebA-HQ dataset (30k face portraits) [14] or LSUN (10-scene and 20-object categories with at least 125k images in each category) [17]. Key research in medical imaging, however, is often carried out on three-dimensional data (3D volumes). Compared to two-dimensional data (2D images), which allows a more precise interpretation of the objects of interest by exploiting their 3D structure and information. The number of voxels is typically much higher than the number of pixels in the two-dimensional counterparts, and processing 3D networks becomes a major challenge. In addition, the lack of large amounts of patient data further limits the applicability of state-of-the-art generative 2D models to the 3D case.

### 1.2. Case Example: 3D Bone-Image Synthesis

**Figure 1:**
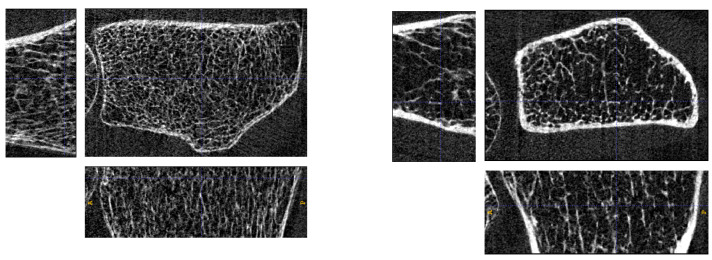
HR-pQCT bone samples of real patients with isotropic voxel size 60.7 μm. Volumes are cropped to a region of interest (ROI) with varying numbers of voxels for each scan.

An example highlighting the need for 3D generative models is the analysis of bone micro-architecture structure. High-resolution peripheral quantitative computed tomography (HR-pQCT) is a 3D medical imaging technique capable of examining in vivo microscopic bone structures in the extremities. Since its introduction in 2005 [18], its use in clinical research into bone-related pathologies has grown rapidly due to the unprecedented resolution of the images [19]. With 3 μSv to 5 μSv effective radiation dose per scan, HR-pQCT is also beneficial to patients compared to conventional (diagnostic) bone-imaging techniques such as dual-energy X-ray absorptiometry (DXA) while providing significantly more valuable information about overall bone quality (image sample Figure 1) [20].

Despite the clear advantages, the current use of HR-pQCT remains confined to research applications. Major obstacles to its adoption into a clinical diagnostic routine are the time-consuming segmentation process [21,22] and the large number of interdependent parameters generated by bone morphometric analysis [20]. Both issues have been addressed using machine learning as detailed in recent publications [20,22]. However, to our knowledge, all existing large cohorts of patient data have been recruited for the study of bone-related pathologies (see [23] as an example). This limits researchers attempting to train and verify their models with HR-pQCT volumes of bones from young, non-pathological patients to small datasets consisting of structures from only a few individuals.

### 1.3. Main Contributions

The results of this work provide a pathway to overcome such limitations by generating arbitrary amounts of 3D volumes with a tailored set of relevant properties. It bridges the gap between recent advances in two-dimensional generative modeling and their implementation for high-resolution 3D medical volumes. To this end, the techniques of progressive growing (ProGAN) [14] and style-based generation (StyleGAN) [15] are extended to the 3D case. The GAN model and the entire training algorithm are developed from scratch in PyTorch (https://pytorch.org/ (accessed on 25 October 2024)).

It is important to note that this work is not just a transfer of the well-established 2D methods ProGAN and StyleGAN to the 3D case. Although the network architecture is mainly based on the two-dimensional counterparts, this paper adds additional analyses: How must 3D media data with variable shapes be prepared to form a three-dimensional dataset for GAN training? What is a good hyperparametric replacement to ensure convergence of network training on comparatively small medical datasets? What latent attribute analysis methods are applicable to 3D data, and how can they be effectively used to process or augment medical data? This work aims to answer all these questions by embedding classical ProGAN and StyleGAN methods in a completely new environment. Alternative methods would also be diffusion models [24,25], as they have been established as a powerful alternative to GANs for learning data distribution on very large datasets. It would be very interesting for future research to investigate diffusion models for medical data synthesis in limited data settings.

Our approach is implemented on a modest sample set of 404 bone volumes obtained through HR-pQCT. The result is a powerful, high-resolution bone-image synthesis model of surprisingly good quality and diversity. Specifically, 64 synthetic instances are assessed by two CT imaging experts. In addition, advanced visual assessment metrics taken from computer vision are implemented and compared to the expert assessment of the generated bone images. This can be used as an expert-driven indicator of how a computer best mirrors human visual perception.

To gain a more detailed understanding of the structure of the 3D model, the latent codes of the model are examined in further detail. Specific attributes of the data and the corresponding latent inputs are used to learn directions in latent space that describe these attributes well. In addition, GAN inversion techniques and latent code manipulation are explored to synthesize customized high-dimensional medical images for attribute-driven data augmentation. The results are supported by many visualizations in the results section, which also includes links to demonstration videos of the proposed analysis of 3D generative models. To ensure reproducibility, exact details on the optimization process and the network architectures are summarized in the Appendix A. An extensive literature search revealed that this is the first work on generating highly detailed bone micro-architecture in 3D. Furthermore, this is the only work to date that investigates latent space properties and automated realism assessment in 3D medical applications.

## 2. Background

### 2.1. Generative Adversarial Networks

In basic terms, a generative adversarial model learns a link function between a low-dimensional latent distribution and a high-dimensional data distribution. The GAN architecture [7] is composed of a generator function G:Z→X and an adversarial counterpart f:X→[0,1]. The elements of latent space Z are commonly assumed to follow a standard normal distribution, i.e., the generator takes a sample z∈Z,z∼N(0,1) and maps it to image space X. The generative function *G* is approximated by a neural network by adaptation of its parameters so that the output distribution of *G* assimilates the distribution of the given training set. Simultaneously, the adversarial function *f* is optimized to distinguish between generated and real instances. In a two-player min–max game, generator parameters are updated to fool a steadily improving discriminator [10]. Already, the initial versions of GANs raised significant interest in the computer vision community but proved to be unstable due to the problem of vanishing gradients and mode collapse. Improving the optimization objective of the generative and adversarial function yielded highly successful modifications of the simple two-player game, like Least-Squares-GAN [26], Spectral-Normalization-GAN [27] or Wasserstein-GAN (WGAN) [28,29]. In particular, the WGAN approach had a crucial impact on training controllability and substantially shaped GAN development. Instead of classifying if a sample is real (f≈1) or has been drawn by a neural network (f≈0), Wasserstein GANs use a new adversarial critic f:X→R to approximate the distance between the real and the generator distribution.

### 2.2. High-Resolution Synthesis

The desire to draw synthetic images in higher resolutions led to the introduction of progressive GAN (ProGAN) [14], which uses a growing strategy for the network training process. The core concept is to start with low resolution for both generative and adversarial functions and then add new layers as training progresses, modeling fine high-frequency details [16]. ProGAN improved both the optimization speed and the stability, facilitating image generation at a resolution of $1024^{2}$ pixels. Controlling the style of synthetic images became increasingly important and has been successfully assessed by style-based GAN (StyleGAN) [30]. The model manipulates mean and variance per channel after each convolution in the generative function to control the style of the output effectively and, similar to ProGAN, enables generation up to a scale of $1024^{2}$ pixels. Improvement of perceptual quality was achieved in StyleGAN2 [15] by including weight demodulation, path length regularization, and network architecture redesign. Embedding adaptive discriminator augmentation in StyleGAN2-Ada [31] yielded reasonable training of style-based generators also on limited datasets. The latest progress has been made in StyleGAN3 [32], which proposed a new architecture to tackle aliasing effects during image transition.

### 2.3. GAN Inversion

ProGAN and StyleGAN enable a meaningful link between image space and corresponding latent and style vectors, respectively. Beside the unconditioned generation of images, these models may also be used for semantic manipulation and effective augmentation of existing data. GAN inversion aims to invert a given instance from data space back into its latent or style representation so that the image can be reconstructed from the inverted code by the pre-trained generative function. GAN inversion plays a critical role in bridging the real and synthetic data domains, leading to significant advances in this fairly young research area [16,33,34,35]. So far, the rapidly growing set of solutions for GAN inversion has been divided into three sub-areas.

Learning-based inversion is characterized using an additional encoding neural network that predicts the latent code from an existing image such that the GAN-based reconstructed counterpart resembles the original. Optimization-based methods directly minimize a pixel-wise reconstruction loss to find a corresponding latent code for an existing image. The minimization objective is commonly solved by the gradient descent method. Both techniques lead to a quality-to-time trade-off [16]—learning-based methods are generally associated with quality degradation of the reconstruction, while optimization-based methods are time-consuming and strongly depend on the initial value for the minimization algorithm. Therefore, hybrid methods are the most widely adopted methods to date, using an encoder-based latent code as the starting value for the subsequent optimization process.

### 2.4. GANs in Medical Imaging

GAN synthesis and inversion have already been adopted by the medical community, where existing methods for inversion and manipulation are used in specific domains like computed tomography (CT) or magnetic resonance imaging (MRI). In [36], the idea of domain-specific GAN inversion [35] is incorporated to synthesize mammograms constrained on shape and texture for psychophysical analysis on a larger scale. In [5], a StyleGAN is trained on both CT and MRI instances, and it is shown how specific attributes can be targeted in the latent space, enabling powerful methods for guided manipulation and modality transfer. While the previously mentioned works only use 2D slices Hong et al. target entire stacks of images, using a 3D-StyleGAN to synthesize MRI images [37]. Although this work demonstrates StyleGAN adoption to 3D data, the authors limit data dimension to $64^{3}$ voxels—a size that is rarely sufficient in real-life medical studies. Furthermore, no analysis of latent code interpretation and manipulation has been made.

Most closely related to our study is the hierarchical amortized GAN (HA-GAN) proposed in [38]. A hierarchical structure is implemented that simultaneously generates a low-resolution version of the 3D dataset and a randomly selected sub-volume of the high-resolution counterpart. In terms of 3D synthesis at high resolution, this work achieves tremendous performance. However, the semantic meanings of the latent space are explored by implementing two additional regression problems. Furthermore, the authors of HA-GAN do not emphasize advanced feature extraction to model the realism of the generated samples. These aspects clearly distinguish HA-GAN from the study presented here.

## 3. Methods

In the present work, two methods for volumetric synthesis are considered: 3D progressive growing GAN (3D-ProGAN) and 3D style-based GAN (3D-StyleGAN). These methods are described in Section 3.2 and applied to a dataset described in Section 3.1. A short glance at the used visual validation metrics is given in Section 3.3. Section 3.4 includes some important details on model training, and Section 3.5 describes the GAN inversion process.

### 3.1. Data Acquisition and Preprocessing

The dataset used for experimentation was obtained from a study on bone health and fracture healing conducted by the Medical University of Innsbruck in collaboration with the department for trauma surgery at the University Hospital of Innsbruck. Subjects were recruited from patients admitted to the emergency outpatient unit due to a fracture of the distal radius. The resulting cohort has an average age of 53.2 years. The youngest patient was 18, and the oldest was 91. The distribution was 60% female and 40% male. In the course of the study, the fractured and non-fractured radii were scanned at six time points within one year. The non-fractured radii were scanned according to a fixed distance protocol (see [19] for details), approximately 10 mm from the distal end of the bone. The intervals were at the date of admission as well as after one week, three weeks, three months, six months, and 12 months—resulting in six distinct volumes per patient. Only volumes from the non-fractured site were used from 98 patients, 515 3D volumes for the dataset in total. A fifth of the volumes were removed before training due to issues with scanning quality, reducing the volume count further to 404 (cf. Section 3.3).

**Figure 2:**
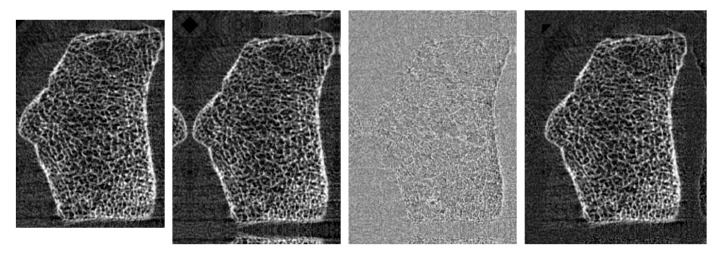
Preprocessing. From left to right: The sample is cropped or padded to a constant size of 168 × 576 × 448 voxels. The mirrored volume is used as padding. The samples are considered regarding the discrete cosine basis. Clipping the basis coefficients to range [−0.001,0.001] yields the noise volume. The padded regions are replaced by the corresponding noise volume.

A strong variation of measured voxels between the individual measurements makes the data-processing a non-trivial task. While 168 axial slices (≈10 mm) were obtained for every sample, the extent in vertical and horizontal direction ranges between [397, 663] and [278, 529] voxels, respectively. The processing pipeline consists of multiple steps and is shown in Figure 2. Each sample is cropped or padded to a constant size of 168 × 576 × 448 voxels. The mirrored image is used as padding, as conventional zero padding is not appropriate in this case due to the high levels of background noise. The samples are considered regarding the discrete cosine basis. Clipping the basis coefficients to range [−0.001, 0.001] yields the noise images. The padded regions are replaced by the corresponding noise image to avoid reflections of the bone itself at the edges. Due to restricted hardware resources, patient data are sub-sampled by factor 2.

Following the described preprocessing pipeline, training data are transformed to a unique shape of 84 × 288 × 224 with constant voxel spacing. To further enlarge the dataset, each scan is divided into four overlapping slice stacks of size 32 × 288 × 224. This is followed by rotations and zoom-in operations using angles in [−10,10] and zoom factors in [1,1.15], both uniformly chosen at random. Using the augmentation pipeline described above, nearly 6800 training instances are obtained from the 404 volumes considered.

### 3.2. Architecture

#### 3.2.1. 3D Progressive Growing GAN

**Figure 3:**
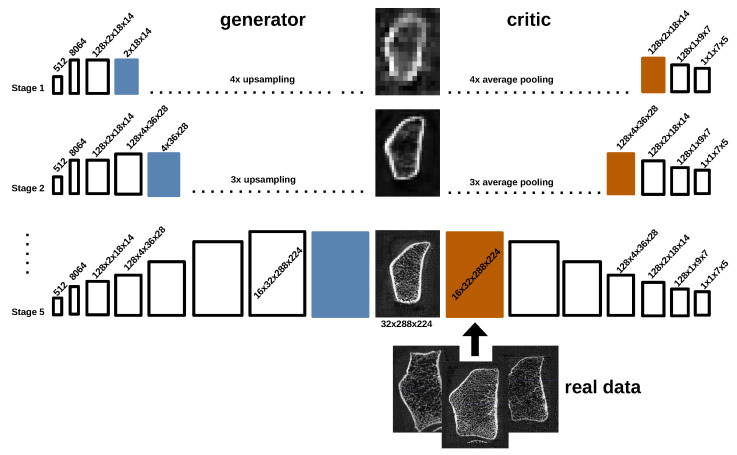
Exemplary visualization of the progressive growing strategy for the synthesis of 3D bone HR-pQCT data.

The generator G:Z→X maps from latent space to image space. To be more precise, a normally distributed latent vector $z\in Z\subset R^{512},\;z∼N\left(\vec{0},\mathrm{Id}\right)$ is sampled and forwarded to a dense layer and a reshape layer with output size $\left[c\;\cdot \;8,d_{1}/32,d_{2}/32,d_{3}/32\right]$, where *c* denotes the channel size of the method and $d_{1},d_{2},d_{3}$ the spatial size of the training data. This is followed by nearest-neighbor upsampling and a block of consecutive 3D convolutional layers. The generator is now called to reside on Stage 1. A repeated application of the same block (upsampling and convolutional block) yields the Stage 2 output. In total, the block is applied 5 times, yielding a final output resolution of $\left[c,d_{1},d_{2},d_{3}\right]$ at Stage 5 (see Figure 3). In Stages 3 to 5, the feature maps are decreased by a factor of 2, yielding channel size *c* at the last stage. Layers shown in blue denote 3D convolution with channel size 1 to transfer learned features to the image domain.

The smooth transition strategy of [14] is applied. As shown in Figure 3, the critic also operates in different stage modes, where the final critic at Stage 5 consists of five strided convolutional layers with increasing channel size and a final output convolutional layer of channel size 1 (cf. PatchGAN [39]). Layers shown in orange denote 3D convolution with channel size *c* to link the image domain with the feature space.

#### 3.2.2. 3D Style-Based GAN

For style-based generation, the generative function can be described by the composition $G=\tilde{G}\circ \Phi :Z\to X$. Similar to 3D-ProGAN, a normally distributed vector $z\in Z\subset R^{512}$ is sampled and then mapped by a mapping network $\Phi :N(\vec{0},\mathrm{Id})\to W$ to a learned intermediate latent space $W\subset R^{512}$ which more faithfully reflects the training data distribution compared to standard normal distribution [33]. The latent code w=Φ(z) is converted to 15 different style codes by learned affine transformations. Incorporating the previously described progressive GAN, these 15 style vectors are fed to the generator $\tilde{G}:W\to X$ using weight demodulation [30], three styles at each stage. After each convolution layer, a noise map is sampled of the same spatial size, scaled by a single learnable parameter and added to each feature map. The critic network for the style-based generator remains unchanged compared to 3D-ProGAN.

For both methods, a video-demonstration of the progressive growing strategy can be viewed online:

- https://www.youtube.com/watch?v=Dicd6cEaZp8 (3D-ProGAN) (accessed on 25 October 2024)
- https://www.youtube.com/watch?v=TbKN0CPWvHE (3D-StyleGAN) (accessed on 25 October 2024)

### 3.3. Validation

To quantitatively evaluate the perceptual quality of intermediate training samples and final results, Frechét Inception Distance (FID) [40] is measured between the distributions of real and synthesized data. FID relies on features extracted from original and synthesized instances, where the feature extractor plays an essential role and should be chosen appropriately for the task. After every image of the generated dataset *A* and real dataset *B* is processed through the chosen feature extractor, the means ($\mu_{A},\mu_{B}$) and covariances ($\Sigma_{A},\Sigma_{B}$) of the extracted features of the two datasets are compared with the following distance:

(1) $$\mathrm{FID}≜{∥\mu_{A}-\mu_{B}∥}_{2}^{2}+\mathrm{tr}\left(\Sigma_{A}+\Sigma_{B}-2{\left(\Sigma_{A}\Sigma_{B}\right)}^{\frac{1}{2}}\right)$$

Higher distances indicate a poorer generative model. A score of 0 indicates a perfect model. This study considers three feature extractors:

1. The originally proposed FID relies on the Inception v3 classification network that was pre-trained on 2D images from ImageNet [41], so this measure is not directly applicable to 3D data. Therefore, from each scan, two axial slices at random positions are selected and used for FID validation. This measure is denoted by ${\mathrm{FID}}_{\mathrm{inc}}$.
2. Similar to HA-GAN [38], a 3D ResNet model pre-trained on 3D medical images [42] is deployed to collect features of the 3D volumes directly. This version is denoted by ${\mathrm{FID}}_{\mathrm{res}}$.
3. Each scan of the 98 patients was evaluated directly after measurement by a medical expert for motion artifacts and given a visual grading score (VGS) score between 1 (best) and 5 (worst), as described by Sode et al. [43] and reiterated by Whittier et al. [19]. Using this rating, a 3D ResNet classifier has been trained. FID using features by the VGS classifier are denoted with ${\mathrm{FID}}_{\mathrm{vgs}}$. Images with a score of 4 or 5 (17% in total) were excluded from GAN training to avoid the network replicating motion artifacts.

The FID has been shown to reflect the human opinion of perceptual quality quite well. However, the FID may also increase when the perceptual quality is sufficiently good, but the synthesis variance is decreasing. Therefore, two additional indicators for synthesis quality are added—precision and recall [44]. Precision quantifies the percentage of generated images that are similar to training data (sufficient perceptual quality), while recall models the percentage of training data that can be recreated by the generator (coverage of the real data distribution). For precision and recall evaluation, only features extracted by the 3D medical ResNet model are considered.

FID, precision, and recall scores compare the distributions of two datasets. Thousands of instances are sampled from both distributions, and corresponding features are used to calculate the scores. Since these are quantitative measures, assessing the plausibility of a single generated sample automatically is not possible and requires human intervention. To evaluate the proposed bone synthesis regarding the measure of realism for single instances, a realism score [44] is adopted. More precisely, the degree of realism increases the closer the features of a generated sample are to the manifold formed by the features of the real training data and decreases otherwise. Similar to FID calculation, three different methods are considered for feature extraction, yielding three different realism scores: $r_{\mathrm{inc}}$, $r_{\mathrm{res}}$ and $r_{\mathrm{vgs}}$. All three feature-extraction methods are compared with the subjective assessment of two human experts on HR-pQCT imaging to determine the realism score that most closely matches human perception.

### 3.4. Training

Similar to [14], Wasserstein loss with a two-sided gradient penalty [28] is deployed to train both the generator and the critic in parallel. Let $P_{X}$ denote the data distribution of bone images, *G* a generator in {3D-ProGAN,3D-StyleGAN} and f:X→R the corresponding critic. Then

(2) $$\begin{array}{cc} \ell_{\mathrm{critic}} & =E_{\begin{array}{c} x∼P_{X}\;\;\;\;\\ z∼N(0,\mathrm{Id}) \end{array}}\left[f\left(G\left(z\right)\right)-f\left(x\right)+p_{1}\;\cdot \;{\left(\left({∥\nabla_{\tilde{x}}f\left(\tilde{x}\right)∥}_{2}-1\right)\right)}^{2}+p_{2}\;\cdot \;f{\left(x\right)}^{2}\right] \end{array}$$

(3) $$\begin{array}{cc} \ell_{\mathrm{generator}} & =E_{z∼N(0,\mathrm{Id})}\left[-f\left(G(z)\right)\right], \end{array}$$

where $p_{1}$ and $p_{2}$ denote the influence of the gradient and drift penalty, respectively. Note that $\tilde{x}$ denotes an arbitrary transition between real and generated domain [28]. The Adam optimizer is used to minimize both objectives in Equations (2) and (3). Optimal architecture and optimizer configurations can be found in Appendix A and Appendix B, respectively. Approximately 10% from the available training data in Section 3.1 was excluded for early detection of critic overfitting during the training process.

### 3.5. GAN Inversion

In order to investigate properties and directions in the latent space, an encoder is built to generate latent codes from existing images, i.e., inverting the generator that has been trained in 3D-ProGAN and 3D-StyleGAN. The encoder has the reversed structure of the generator (cf. Table A1). Pixel feature normalization is removed, and for 3D-StyleGAN inversion, two fully connected layers with leaky ReLU activation are added at the bottom of the encoder. Using a pre-trained generator *G* and the corresponding adversarial critic *f*, the optimization of the encoder $E:X\to R^{512}$ closely follows [33].

For 3D-ProGAN, a hybrid approach is used, i.e., an initial guess for the latent code is obtained by propagation through the learned encoder while refinement of the given code is enabled by a subsequent minimization task. Let $f_{L-1}$ denote the penultimate convolution layer of the adversarial critic *f*. Three loss terms for distortion (*dist*), perceptual similarity (*perc*) and latent code plausibility (*latent*) are defined as follows:

(4) $$\begin{array}{cc} \; & \ell_{\mathrm{dist}}\left(x,E\right)=\frac{0.5}{\#\mathrm{voxel}}\sum_{p}^{\#\mathrm{voxel}}{\left(x_{p}-G{\left(E(x)\right)}_{p}\right)}^{2}, \end{array}$$

(5) $$\begin{array}{cc} \;\; & \ell_{\mathrm{perc}}\left(x,E\right)=\frac{0.5}{\#\mathrm{features}}\sum_{q}^{\#\mathrm{features}}{\left(f_{L-1}{\left(x\right)}_{q}-f_{L-1}{\left(G(E(x))\right)}_{q}\right)}^{2}, \end{array}$$

(6) $$\begin{array}{cc} \;\; & \ell_{\mathrm{latent}}\left(x,E\right)=\frac{1}{1024}\sum_{r=1}^{512}{\left(E{\left(x\right)}_{r}\right)}^{2}. \end{array}$$

The risk function for the encoder *E* and the optimization objective that yields the optimal latent code $z_{\mathrm{opt}}\left(\hat{x}\right)$ for a given image $\hat{x}\in X$ are defined as:

(7) $$\begin{array}{cc} \ell_{\mathrm{encoder}} & =E_{x∼P_{X}}\left[\ell_{\mathrm{dist}}\left(x,E\right)+\ell_{\mathrm{perc}}\left(x,E\right)+\ell_{\mathrm{latent}}\left(x,E\right)\right], \end{array}$$

(8) $$\begin{array}{cc} \;z_{\mathrm{opt}}\left(\hat{x}\right) & =\underset{\begin{array}{c} z\in R^{512} \end{array}}{arg min}\left[\frac{1}{\#\mathrm{vox}}{∥\hat{x}-G\left(z\right)∥}_{2}^{2}+\frac{1}{\#\mathrm{feat}}{∥f_{L-1}\left(\hat{x}\right)-f_{L-1}\left(G(z)\right)∥}_{2}^{2}+\frac{1}{512}{∥z∥}_{2}^{2}\right]. \end{array}$$

During encoder training, the loss function in (7) is minimized using Adam algorithm with hyperparameters $\left(\alpha ,\beta_{1},\beta_{2}\right)=\left(3\times 10^{-3},0.5,0.9\right)$. For the optimization in Equation (8), the Adam algorithm is again used for 100 updates with a learning rate equal to 7 × 10^−3^.

For 3D-StyleGAN, a similar hybrid approach is considered with a modified functional for latent code plausibility. For style-based generation, the latent codes are not assumed to follow a multivariate normal distribution but the sampled vectors are mapped to a learned latent space W by the mapping $\Phi :N(\vec{0},\mathrm{Id})\to W$ and then forwarded to image space by generator $\tilde{G}:W\to X$. Therefore, given a real image, the retrieved latent code should also reside in the learned latent space. Analogous to [33], a latent discriminator $D_{W}:R^{512}\to \left[0,1\right]$ is trained to distinguish between latent codes constructed by the encoder (fake codes) and by the mapping Φ (real codes). The loss functional for latent code plausibility is adapted as follows:

(9) $$\ell_{W}\left(x,E\right)=-\frac{1}{512}\sum_{r=1}^{512}\log\left(D_{W}\left(E(x)\right)\right).$$

In the case of style-based bone synthesis, the risk functional for the encoder *E* and the optimization objective that yields the optimal latent code $z_{\mathrm{opt}}\left(\hat{x}\right)$ are defined as:

(10) $$\begin{array}{cc} \ell_{\mathrm{encoder}} & =E_{x∼P_{X}}\left[5\;\cdot \;\ell_{\mathrm{dist}}\left(x,E\right)+\ell_{\mathrm{perc}}\left(x,E\right)+0.04\;\cdot \;\ell_{W}\left(x,E\right)\right], \end{array}$$

(11) $$\begin{array}{cc} \;w_{\mathrm{opt}}\left(\hat{x}\right) & =\underset{\begin{array}{c} w\in R^{512} \end{array}}{arg min}\left[\frac{1}{\#\mathrm{feat}}{∥f_{L-1}\left(\hat{x}\right)-f_{L-1}\left(\tilde{G}\left(w\right)\right)∥}_{2}^{2}\right]. \end{array}$$

Technical details and parameters for Equations (10) and (11) are the same as for 3D-ProGAN.

## 4. Results and Discussion

### 4.1. Image Quality

**Figure 4:**
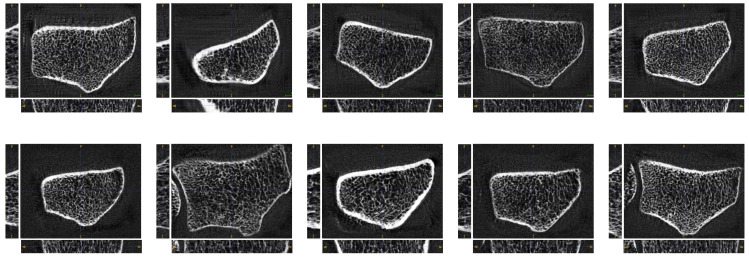
Ten HR-pQCT volumes sampled from the proposed 3D-ProGAN (**first row**) and 3D-StyleGAN (**second row**). Synthesized volumes have spatial size of 32 × 288 × 224.

During training, the data quality of synthesized instances is assessed after every 1000 generator updates via FID, precision, and recall (cf. Section 3.3). Results are represented from Stage 5 with final data resolution 32 × 288 × 224. The truncation trick [14,30] is deployed in Figure 4. For 3D-ProGAN, a truncated normal distribution with truncation level 1.8 is considered for sampling the latent codes. For 3D-StyleGAN, a latent code w∈W is normalized such that $w_{\mathrm{norm}}≔\bar{w}+\psi \;\cdot \;\left(w-\bar{w}\right)$ where $\bar{w}≔E_{z∼N(\vec{0},\mathrm{Id})}\Phi \left(z\right)$ denotes the average latent code and ψ is set to 0.8.

Table 1 summarizes the results for the quantitative validation metrics described in Section 3.3. For both methods, 3D-ProGAN and 3D-StyleGAN, the hyperparameters were determined by a random grid search. More precisely, sets with equidistant values for the channel size of the critic ($c_{c}$), the channel size of the generator ($c_{g}$), the learning rate (α), and the number of critic iterations per generator update ($n_{c}$) were defined and 30 parameter combinations were randomly sampled. For each method, the three winning hyperparameter combinations are summarized in the table. With a ${\mathrm{FID}}_{\mathrm{inc}}$ and ${\mathrm{FID}}_{\mathrm{res}}$ equal to 21.59 and 0.04, respectively, superior performance with respect to those two metrics is achieved by 3D-ProGAN. In terms of ${\mathrm{FID}}_{\mathrm{vgs}}$, 3D-StyleGAN significantly outperforms 3D-ProGAN. Interestingly, 3D-StyleGAN also yields the highest precision, while, in general, higher recall is achieved by 3D-ProGAN.

Indeed, comparing the second row of images (as produced by 3D-StyleGAN) with the first row (3D-ProGAN) in Figure 4 clearly shows superiority regarding perceptual quality for 3D-StyleGAN. It is recommended to view the image enlarged to better observe the high-resolution quality and synthesized high-frequency details.

**Figure A1:**
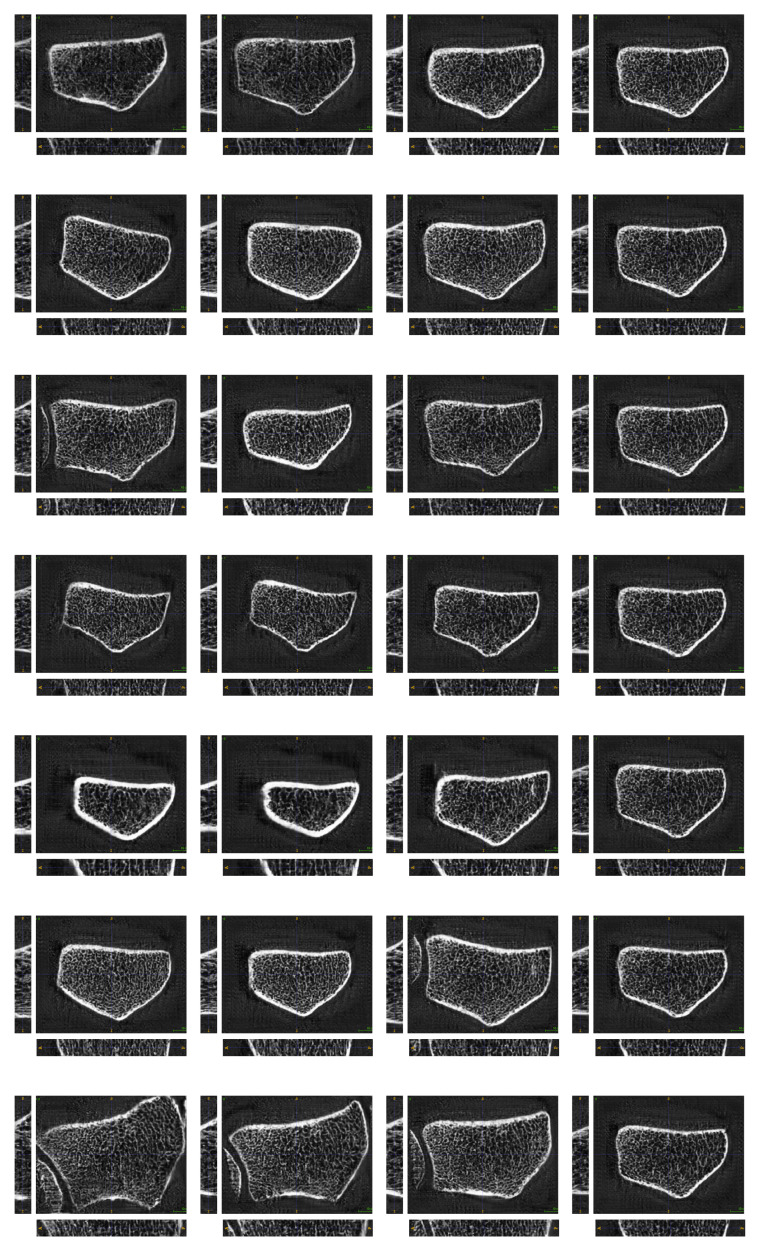
Synthetic HR-pQCT volumes sampled from the proposed 3D-ProGAN approach with varying parameters for the truncated normal distribution. From left to right column: truncation parameter equals {2.6,1.8,1,0.2}.

**Figure A2:**
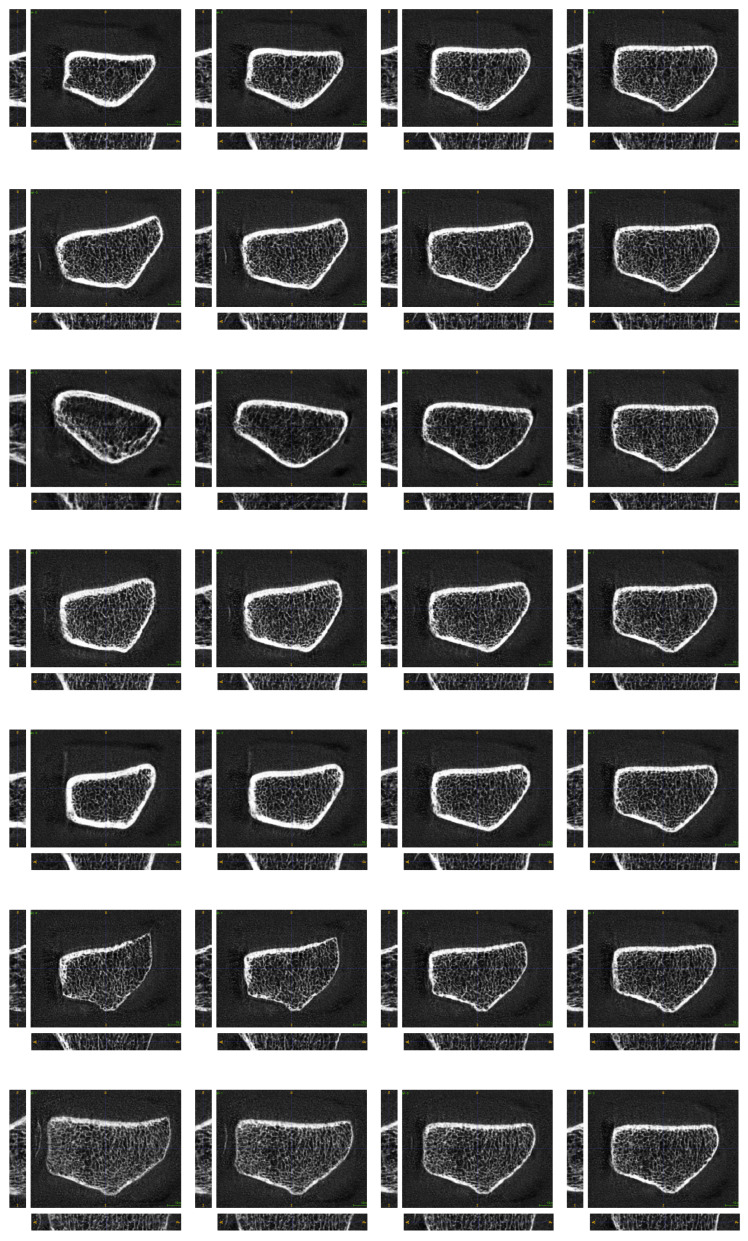
Synthetic HR-pQCT volumes sampled from the proposed 3D-StyleGAN approach with varying truncation levels. From left to right column: ψ={1,0.7,0.4,0.1}.

It should be noted that the validation metric ${\mathrm{FID}}_{\mathrm{res}}$ exhibits rather high variance, especially for the 3D-StyleGAN method. Arguably, due to the noise in the training data and consequently in the generated data, the features extracted by a 3D ResNet pre-trained on medical data [42] may not be representative. Further samples with varying truncation levels are displayed in Figure A1 and Figure A2 (see Appendix C).

During the evaluation process, a graphical user interface was implemented. The use of the GUI for truncation-triggered data synthesis and download is visualized in short demo videos:

- https://www.youtube.com/watch?v=K8UbsFTSaqE (3D-ProGAN) (accessed on 25 October 2024)
- https://www.youtube.com/watch?v=4VPDUZ3Pbk8 (3D-StyleGAN) (accessed on 25 October 2024)

### 4.2. Image Transition

The previous section demonstrates the ability to successfully generate high-resolution bone CTs with high diversity. Generation by sampling latent codes can be used to extend datasets in an unconditioned manner. In this case, the distribution of a given attribute in the synthesized data is very likely to follow the distribution of the same attribute in the training set. In this section, a method is proposed for synthesizing data with respect to a particular attribute.

Image transition aims to semantically interpolate two medical samples by propagating a weighted sum of the corresponding latent codes through a fixed generative function. This is suitable for investigating the plausibility of the inverted codes—for a good GAN inversion, the spatial and semantic attributes should vary continuously during the transition from one inverted code to the other inverted counterpart. If the underlying scans of both codes share a certain attribute, all generated scans during the transition should also share this attribute.

For investigation, two specific properties of bone HR-pQCT data are targeted—trabecular bone mineral density (Tb.BMD) and cortical bone mineral density (Ct.BMD). Ct.BMD and Tb.BMD corresponds to the average mineral density (i.e., X-ray beam attenuation) within the voxel volume of the cortical and trabecular compartments, respectively, and is calculated directly from the grayscale image data [19]. These attributes have been shown to be statistically linked to bone fracture risk [20]. As the training data are comprised of images from patients who experienced a bone fracture, the distribution of Ct.BMD and Tb.BMD values in the dataset are not normally distributed, exhibiting a slight bias.

**Figure 5:**
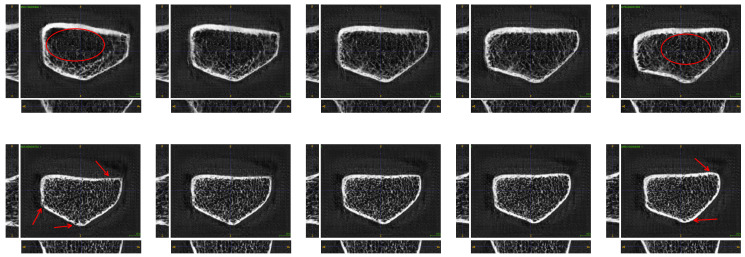
**First row**: samples with weak trabecular bone mineralization (Tb.BMD). **Second row**: samples with weak cortical bone mineralization (Ct.BMD). From left to right: $x_{1},\;x_{1,2}^{0.25},\;x_{1,2}^{0.5},\;x_{1,2}^{0.75},\;x_{2}$. The areas marked in red allow the reader to better recognize the low Tb.BMD and the weak Ct.BMD of the examined radii, respectively.

Let $x_{1},x_{2}\in X$ denote two samples from the training set with a small value for Tb.BMD. The GAN inversion strategy discussed in Section 3.5 is applied for 3D-ProGAN. According to Equation (8), this yields $z_{1}≔z_{\mathrm{opt}}\left(x_{1}\right)$ and $z_{2}≔z_{\mathrm{opt}}\left(x_{2}\right)$. During transition, the generative function *G* of 3D-ProGAN is used to generate new samples $x_{1,2}^{\alpha }=G\left(\alpha \;\cdot \;z_{1}+\left(1-\alpha \right)\;\cdot \;z_{2}\right)$ for α∈[0,1]. In Figure 5, the generated results are displayed in the first row. Obviously, the average bone mineralization in the trabecular compartment is weak for all scans, while a smooth spatial transition from $x_{1}$ to $x_{2}$ can be observed. The second row shows the same procedure repeated with samples for $x_{1}$ and $x_{2}$ exhibiting small Ct.BMD values. The implemented GUI provides an interactive way to use the image transition to synthesize new data for augmentation. A demonstration video can be found here: https://www.youtube.com/watch?v=j6Fh0a4r1Rw (accessed on 25 October 2024).

### 4.3. Style Mixing

The interpolation between scans discussed above allows for a smooth transition between different shapes while preserving certain attributes. However, it is also possible to fix a certain property of the first patient (e.g., shape) and mix it with the given style of a second patient (e.g., trabecular properties). The 3D-StyleGAN allows the manipulation of the output of the generative function using the style transfer capability of the network, where two latent codes of the learned latent space W are included in the generation process. As described in Section 3.2, a latent code *w* is converted by learned affine transformations into 15 different style codes, which are fed into the generative function using weight demodulation. The idea of style mixing is to feed the style codes based on the source scan and the codes from the target scan to the generator.

Let s∈X and t∈X denote the source and target images of real patients, respectively. Applying the GAN inversion strategy in Section 3.5 for 3D-StyleGAN yields $w_{s}≔w_{\mathrm{opt}}\left(s\right)$ and $w_{t}≔w_{\mathrm{opt}}\left(t\right)$, where both inverted codes are forced to reside in W by the latent discriminator (cf. Equation (9)). 3D-StyleGAN consists of a generator $\tilde{G}:W^{15}\to X$ that takes 15 different style vectors based on latent input *w* and feeds them to the convolutional layers by weight demodulation [30]. Variation of different styles is enabled using style vectors based on both latent source code $w_{s}$ and latent target code $w_{t}$. Let $x_{s,t}^{a}$ denote a generated sample of 3D-StyleGAN that used style vectors of $w_{s}$ for the first *a* convolution layers and style vectors of $w_{t}$ for the remaining convolution layers.

**Figure 6:**
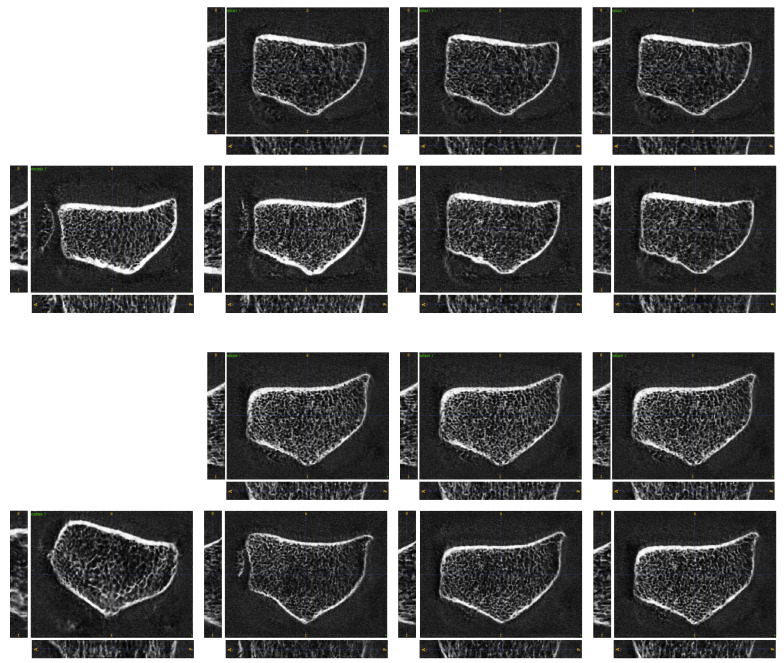
An illustration of the style combination based on the 3D-StyleGAN approach. For both examples, the first row denotes the source image (real patient data). The second row contains the target image at the left most position and style mix results where the style of the source is fed to the generator in the first three convolutional layers ($x_{s,t}^{3}$), in the first seven layers ($x_{s,t}^{7}$) and in the first twelve layers ($x_{s,t}^{12}$), from left to right.

Figure 6 shows sample results for this technique. The top-most row shows the same source image three times, taken from a patient with a comparatively low Ct.BMD value. The second row displays the target image with a high Ct.BMD value as well as the style mix results $x_{s,t}^{3}$, $x_{s,t}^{7}$ and $x_{s,t}^{12}$. It can be observed that $x_{s,t}^{3}$ yields an interpolation of both shapes and a strong cortical bone structure. Increasing the number of source style vectors to seven in $x_{s,t}^{7}$ yields a bone with a similar shape to the source but with the cortical property of the target. This is an essential result for this study—it is possible to apply a certain attribute from a target image to the shape of another source. Only using three style vectors of the target scan in the last three convolution layers ($x_{s,t}^{12}$) yields nearly no differences from the source scan.

The third and fourth rows of Figure 6 show the mixed approach, repeated for trabecular bone mineral density. Again, $x_{s,t}^{3}$ shows a transition between both shapes with small Tb.BMD value, $x_{s,t}^{7}$ yields a copy of the source image with significant changes in the trabecular structure, and $x_{s,t}^{12}$ is quite similar to the source image.

In conclusion, the use of the proposed 3D-StyleGAN for style mixing appears to be another reliable tool for editing HR-pQCT attributes. It can be concluded that styles applied to low-resolution convolution layers determine spatial attributes of the bone, while codes applied to higher-resolution layers are responsible for variations in semantic features such as cortical or trabecular conditions.

### 4.4. Attribute Editing

The previous section demonstrated the impact of the latent representation on different resolutions in the generative function. To complete the analysis of the relationship between latent and image space, the following section examines the interpretability of latent space. In two-dimensional applications, generative networks have been shown to automatically learn to represent multiple interpretable attributes in latent space [16,34,45]. These works suggest the identification of a semantically meaningful direction $n\in R^{512}$ in order to achieve a manipulation $x_{\mathrm{edit}}=G\left(z_{\mathrm{opt}}\left(x\right)+\alpha n\right)$.

According to an extensive literature survey, this is the first study to leverage the exploration of meaningful directions to the 3D case in an unsupervised manner. The approach in [34] is used to find the optimal direction $n^{*}$:

(12) $$n^{*}=\underset{\left\{n\in R^{512}|n^{T}n=1\right\}}{arg max}{∥An∥}_{2}^{2}.$$

The matrix *A* denotes either the first linear layer in 3D-ProGAN or the concatenation of 15 linear layers in 3D-StyleGAN, which converts the latent code into a style code. The term optimal directions correspond to a vector that causes large variations after projection by *A*. Similar to [34], the top four directions $n_{1},n_{2},n_{3},n_{4}$ are determined using the eigenvectors of $A^{T}A$ associated with the four largest eigenvalues.

**Figure 7:**
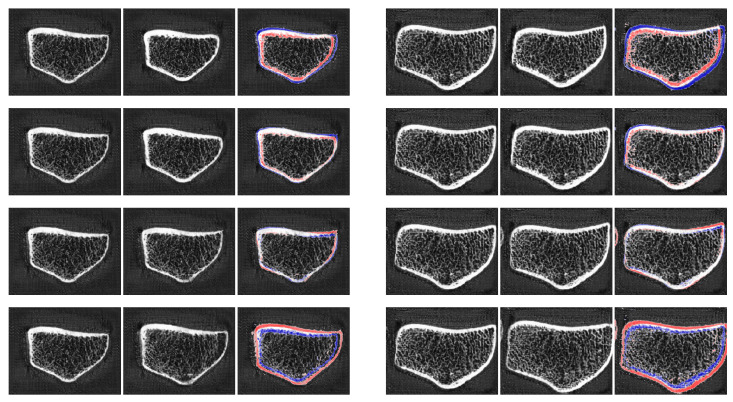
3D-ProGAN results for attribute editing. For each volumetric sample, the center axial slice is visualized. Left: Existing patient *x*. Middle: Generated samples $G_{1}\left(z_{\mathrm{opt}}\left(x\right)+\alpha n_{k}\right),\;k=1,2,3,4$. Right: difference $G_{1}\left(z_{\mathrm{opt}}\left(x\right)+\alpha n_{k}\right)-x$, where red and blue voxels denote positive and negative residuals, respectively.

Figure 7 shows the latent space analysis applied to 3D-ProGAN. A subsequent analysis of the manipulated images is necessary to understand which property each direction $n_{1},n_{2},n_{3},n_{4}$ encodes. The first direction $n_{1}$ shrinks the circumference of the cortical compartment while leaving semantic properties unchanged (first row). $n_{2}$ significantly enlarges the cortical compartment (second row). The third vector $n_{3}$ results in a slight rotation of the bone, while $n_{4}$, complementary to $n_{1}$, enlarges the circumference of the cortical compartment (third and fourth row). In all editing operations, the strength of manipulation α equals 4. All four directions may be used in data-augmentation scenarios to increase bone size, change the cortical thickness, or rotate the sample. Interestingly, none of the four latent directions has a crucial impact on the trabecular properties. These may be varied using an eigenvector associated with smaller eigenvalues.

### 4.5. Expert Validation

**Figure 8:**
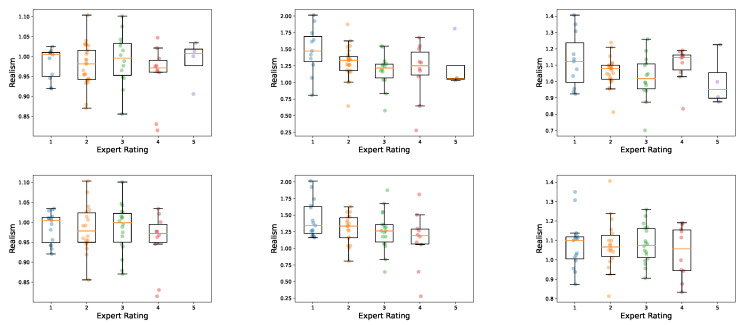
Comparison between computer-based realism scores and the subjective rating by Expert 1 (**first row**) and Expert 2 (**second row**) on HR-pQCT images. The horizontal axes denote the expert rating 1–5, while the vertical axes show the calculated realism scores. From left to right: $r_{\mathrm{inc}}$, $r_{\mathrm{res}}$, $r_{\mathrm{vgs}}$.

An essential research goal for this work is to investigate computer-based metrics and their ability to approximate the visual perception of human experts in the field. As already thoroughly discussed in Section 3.3, three realism scores, based on three different feature-extraction methods, are utilized: $r_{\mathrm{inc}}$, $r_{\mathrm{res}}$, and $r_{\mathrm{vgs}}$. In contrast to the Frechét Inception Distance, which measures the distance between distributions quantitatively, these realism scores enable the evaluation of perceptual quality for a single sample. These metrics are evaluated using 64 synthetic volumetric images generated using the 3D-ProGAN method. These examples were also evaluated by two CT imaging experts, focusing in particular on image sharpness, valid image area, artifacts, contours, and repetitive patterns in the trabecular structure. Based on these criteria, a score of 1 to 5 was assigned, with a lower score indicating a better rating. The results are depicted in Figure 8.

No clear correlation can be found between the expert’s opinion and the realism score based on the Inception v3 classification network $r_{\mathrm{inc}}$. This is not the case for $r_{\mathrm{res}}$ and $r_{\mathrm{vgs}}$. Both realism scores can distinguish between low and high expert-rated samples to some extent. Especially for $r_{\mathrm{res}}$, which was generated using a 3D ResNet model pre-trained on medical data for feature extraction, the correlation is quite clear for Experts 1 and 2. However, none of the considered realism metrics can accurately reflect the subjective opinion of a human expert. Evaluation of a larger synthetic cohort, involvement of more experts, and a wider range of feature-extraction methods will be part of future research.

## 5. Conclusions and Future Impact

This work demonstrates that three-dimensional generative models can be successfully trained to generate high-resolution medical images of fine-detailed micro-architectures on a voxel basis. In particular, progressive growing and style-based GAN architectures were shown to be viable for the synthetic creation of realistic volumetric grayscale images. Furthermore, GAN inversion techniques are used to map measurable image attributes to directions in a low-dimensional latent space, which allows generated images to be parameterized regarding those attributes. Considering style-based generation, it is possible to mix the characteristics of two source images, creating realistic results that combine selected properties in a controllable manner. Given the modest number of images used in training when compared with the volumes used for similar (2D) image generator networks, the results are definitely impressive. While tell-tale artifacts in the background noise are easily spotted by human experts, the overall structure and small-scale details of the generated bones closely follow the natural patterns. Regarding naturalism, the variation of the shape outlines is, in general, very realistic and shows great variability.

Naturally, this work still has some limitations. For one, the cohort, even if it shows a high diversity in age and gender, represents only the central European population. However, a more generalized model could be derived using the same method by increasing the sample size and adding cohorts from other research groups. The main difficulty in increasing heterogeneity is the availability of free shareable datasets or the formalizing of cooperation agreements. As our main goal is a feasibility study, we decided to use locally available data. Furthermore, the implementation of an automated realism assessment that mimics the perception of human experts mainly depends on an appropriate feature-extraction method. While this study has shown that commonly used feature-extraction models only approximate human perception to a certain extent, appropriate feature computation still requires further research. While an automated realism score would be greatly helpful for large batch image-generation jobs, it does not impact the overall usefulness of the generative models. It should also be noted that the resolution of the generated images, while already high for the standards of generative models, is still below that of original HR-pQCT scans. However, this could be overcome using a hierarchical method that, at least for the high-resolution stages, generates only a subset of slices instead of the entire image.

Regarding the applications for research, the ability to synthetically generate realistic, parameterized medical images from a comparatively small set of originals has great potential for enabling algorithmic research. The example at hand is particularly useful to illustrate the possible advantages: as already stated in the introduction, HR-pQCT has well-documented advantages over current gold-standard diagnostic bone-imaging modalities (i.e., DXA) regarding the resolution and information to be gained from the imaging. Due to current usage being limited to research applications, obtaining sizeable cohorts of images with a distribution that reflects the average population, especially in younger age groups, can be challenging. However, such cohorts are invaluable for the assessment of potential algorithms for diagnostic and processing applications. While the use of fully synthetic datasets for algorithm training may pose other risks, there are multiple scenarios where the augmentation of sample volumes with generated data can be a great advantage. For instance, the ability to customize image attributes may be used to synthesize an optimally distributed test set. The mixing of style-based properties, on the other hand, may be used as a novel form of data augmentation for small datasets, with the ability to generate unique images that show a much larger variance than would be possible with conventional (affine) augmentation techniques. As the ability to rapidly implement graphical user interfaces enables easy adoption by non-expert users, the number of novel uses for image-generation techniques can be expected to rise exponentially in the future.

## Figures and Tables

**Table 1:** Quantitative results for different hyperparameter settings. Considered hyperparameters are truncation level (tr), channel size of the critic ($c_{c}$), channel size of the generator ($c_{g}$), learning rate (α), and number of critic iterations per generator updates ($n_{c}$) (minimal values are highlighted in bold).

tr	$c_{c}$	$c_{g}$	α	$n_{c}$	${\mathrm{FID}}_{\mathrm{inc}}$	${\mathrm{FID}}_{\mathrm{res}}$	${\mathrm{FID}}_{\mathrm{vgs}}$	prec	rec
**3D-ProGAN**									
5	16	20	4 × 10^−3^	5	23.54	0.044	0.182	0.91	**0.91**
1.8	16	20	4 × 10^−3^	5	23.39	0.045	0.233	0.95	0.86
5	12	20	4 × 10^−3^	5	25.98	0.080	0.333	0.94	0.90
1.8	12	20	4 × 10^−3^	5	27.05	0.044	0.454	0.96	0.83
5	20	20	3 × 10^−3^	7	**21.59**	**0.040**	0.219	0.95	0.86
1.8	20	20	3 × 10^−3^	7	23.31	0.259	0.274	0.97	0.82
**3D-StyleGAN**									
1	16	20	4 × 10^−3^	6	26.29	1.478	0.157	0.94	0.89
0.8	16	20	4 × 10^−3^	6	28.99	1.343	0.258	**0.98**	0.78
1	16	16	2 × 10^−3^	6	25.91	0.198	0.329	0.93	0.86
0.8	16	16	2 × 10^−3^	6	28.11	0.883	0.571	0.97	0.75
1	16	20	4 × 10^−3^	5	26.32	0.290	**0.151**	0.93	0.85
0.8	16	20	4 × 10^−3^	5	29.07	0.509	0.206	0.96	0.70

## Data Availability

The training image data used in this project is patient related and therefore can only be shared with permission of the ethical board of the Medical University on special request.

## Abbreviations

The following abbreviations are used in this manuscript:

CT	computed tomography
DL	deep learning
DXA	dual-energy X-ray absorptiometry
FID	Frechet Inception Distance
GAN	generative adversarial network
HA-GAN	hierarchical amortized generative adversarial network
HR-pQCT	High-resolution peripheral quantitative computed tomography
MRI	magnetic resonance imaging
ProGAN	progressive growing generative adversarial network
StyleGAN	style-based generative adversarial network
VGS	visual grading score
WGAN	Wasserstein generative adversarial network
Tb.BMD	trabecular bone mineral density
Ct.BMD	cortical bone mineral density

## Appendix A. Generator Configurations

**Table A1:** Generator details for 3D-ProGAN with method channel size equal to 16. For each layer, information on output channel size (o.c.s), input layer (in) and corresponding activation function (activ.) is provided. Each convolution layer except *out* is followed by a pixel-wise feature normalization [14].

	Type	o.c.s.	in	Activ.
d1	Dense	8064	*z*	
r1	Reshape	128	d1	
u1	Upsample	128	r1	
c11	Conv3 × 3 × 3	128	u1	swish
c12	Conv3 × 3 × 3	128	c11	swish
u2	Upsample	128	c12	
c21	Conv3 × 3 × 3	128	u2	swish
c22	Conv3 × 3 × 3	128	c21	swish
u3	Upsample	128	c22	
c31	Conv3 × 3 × 3	64	u3	swish
c32	Conv3 × 3 × 3	64	c31	swish
u4	Upsample	128	c32	
c41	Conv3 × 3 × 3	32	u4	swish
c42	Conv3 × 3 × 3	32	c41	swish
u5	Upsample	128	c42	
c51	Conv3 × 3 × 3	16	u5	swish
c52	Conv3 × 3 × 3	16	c51	swish
out	Conv3 × 3 × 3	1	c52	sigmoid

**Table A2:** Generator details for 3D-StyleGAN with method channel size equal to 16. For each layer, information on output channel size (o.c.s), input layer (in), and corresponding activation function (activ.) is provided. The input layer *cc* denotes a constant layer, where the scale is a learned parameter.

	Type	o.c.s.	in	Activ.
m1	Dense	512	*z*	LReLU
m2	Dense	512	m1	LReLU
⋮	⋮	⋮	⋮	⋮
*w*	Dense	128	m5	LReLU
s1	Dense	128	*w*	
s2	Dense	128	*w*	
⋮	⋮	⋮	⋮	
s15	Dense	16	*w*	
c11	Demod3 × 3 × 3	128	cc, s1	
c12	Noise	128	c11	LReLU
up1	Upsample	128	c12	
c13	Demod3 × 3 × 3	128	up1, s2	
c14	Noise	128	c13	LReLU
c15	Demod3 × 3 × 3	128	c14, s3	
c16	Noise	128	c15	LReLU
c21	Demod3 × 3 × 3	128	c16, s4	
c22	Noise	128	c21	LReLU
up2	Upsample	128	c22	
c23	Demod3 × 3 × 3	128	up2, s5	
c24	Noise	128	c23	LReLU
c25	Demod3 × 3 × 3	128	c24, s6	
c26	Noise	128	c25	LReLU
⋮	⋮	⋮	⋮	⋮
c51	Demod3 × 3 × 3	16	c46, s13	
c52	Noise	16	c51	LReLU
up5	Upsample	16	c52	
c53	Demod3 × 3 × 3	128	up5, s14	
c54	Noise	16	c53	LReLU
c55	Demod3 × 3 × 3	128	c54, s15	
c56	Noise	16	c55	LReLU
out	Conv3 × 3 × 3	1	c56	sigmoid

## Appendix B. Training Details

The generators in 3D-ProGAN and 3D-StyleGAN have been trained using Adam optimizer with hyperparameters $\beta_{1}=0,\;\beta_{2}=0.98,\;\epsilon =1\times 10^{-7}$ and different learning rates $\alpha \in \{k\times 10^{-3}|k=2,\ldots ,6\}$. Gradient norm clipping with threshold 2 is applied at each step. The concept of equalized learning rates is used. Thus, all convolution and dense layers are initialized using the standard normal distribution. For 3D-StyleGAN, the learning rates of the latent space mapping network Φ are multiplied by factor 0.02.

The critic networks have been trained using Adam optimizer with hyperparameters $\beta_{1}=0,\;\beta_{2}=0.98,\;\epsilon =5\times 10^{-5}$ and different learning rates $\alpha \in \{k\times 10^{-3}|k=2,\ldots ,6\}$. One generator update is followed by $n_{c}$ critic updates, where $n_{c}\in \left\{5,6,7,8\right\}$ during the experiments. Training at Stage 1 is continued until the critic has seen 180k samples. Training on Stage 2 is conducted for 360k scans, while transition of the new layers takes place for the first 180k samples. The procedure is continued until the model reaches final Stage 5. Every time a new stage is reached, the learning rates are multiplied by a factor of 0.85. The size of the minibatches for Stages 1 to 5 equals 24, 24, 12, 6, 3. Training lasts approximately two days on an NVIDIA A100 40 GB GPU.

## Appendix C. Further Visualizations

In the following, more synthetic HR-pQCT instances sampled from both proposed methods, 3D-ProGAN and 3D-StyleGAN, are visualized. Each column represents a different level of truncation. For both generation methods, greater use of the truncation trick increases image quality but at the cost of reduced synthesis diversity.
